# A quantum chemical molecular dynamics repository of solvated ions

**DOI:** 10.1038/s41597-022-01527-8

**Published:** 2022-07-21

**Authors:** Kasimir P. Gregory, Gareth R. Elliott, Erica J. Wanless, Grant B. Webber, Alister J. Page

**Affiliations:** 1 Discipline of Chemistry, School of Environmental & Life Sciences, University of Newcastle, Callaghan, NSW 2308 Australia; 2 Department of Materials Physics, Research School of Physics, Australian National University, Canberra, ACT 0200 Australia; 3 Discipline of Chemical Engineering, School of Engineering, University of Newcastle, Callaghan, NSW 2308 Australia

**Keywords:** Biophysical chemistry, Molecular dynamics, Batteries, Thermodynamics

## Abstract

The importance of ion-solvent interactions in predicting specific ion effects in contexts ranging from viral activity through to electrolyte viscosity cannot be underestimated. Moreover, investigations of specific ion effects in nonaqueous systems, highly relevant to battery technologies, biochemical systems and colloid science, are severely limited by data deficiency. Here, we report IonSolvR – a collection of more than 3,000 distinct nanosecond-scale *ab initio* molecular dynamics simulations of ions in aqueous and non-aqueous solvent environments at varying effective concentrations. Density functional tight binding (DFTB) is used to detail the solvation structure of up to 55 solutes in 28 different protic and aprotic solvents. DFTB is a fast quantum chemical method, and as such enables us to bridge the gap between efficient computational scaling and maintaining accuracy, while using an internally-consistent simulation technique. We validate the database against experimental data and provide guidance for accessing individual IonSolvR records.

## Abstract (summary)

**Table:** 

Measurement(s)	solvation structure
Technology Type(s)	quantum chemistry computational method • Molecular Dynamics

## Background & Summary

Solvated ions are key to myriad processes spanning chemistry, biology, environmental and geophysical systems. Indeed, life as we know it is determined by the way in which ions interact with solvents and other dissolved solutes. In part this is because different ions yield different physicochemical phenomena, so-called specific ion effects (SIEs)^1^. For instance, fluoride and iodide anions can respectively increase and decrease the activity of human rhinovirus and HSV-1 (usually responsible for the common cold and cold sores, respectively)^2,3^. The effect of the anion here is obviously specific to its identity.

Despite being more than 130 years since SIEs were first observed, consensus regarding their origins has not yet been reached. Early theories^4^ were based on an ion’s effect on the surrounding solvent structure, with a particular focus on water. A number of recent studies however have shown that SIEs occur in nonaqueous solvents^5–13^, and many SIEs appear to be quantifiable without directly considering the solvent whatsoever^14^. Nevertheless, structural considerations of the solvent are evidently required when considering bulk electrolyte properties, such as solvation enthalpies and solution viscosities^14^. A primary impediment here is the lack of self-consistent data concerning the way in which ions and solvents interact. For instance, while experimental techniques (e.g., X-ray and neutron diffraction, spectroscopic methods)^15–18^ can probe ion solvation structure, studies are typically limited to a handful of different ions, solvents or concentration ranges. In some cases, ion solvation structure can only be inferred indirectly from experimental data (e.g., electrostriction)^19^. On the other hand, theoretical simulation techniques such as molecular dynamics (MD) can directly probe ion solvation structure (e.g., via radial distribution function, diffusion rates, coordination numbers, etc.), delivering detailed insight into ion solvation structure in some cases^20–32^. The principal limitation with classical MD however is the variability in simulation parameters, such as the MD force field, ensemble, time integration algorithm etc. Importantly, as MD force fields are typically parameterised with a specific (or small number) of physical systems in mind, they often have limited transferability between systems and solvents. While transferability is less of an issue for *ab initio* molecular dynamics (AIMD) or hybrid quantum mechanics/molecular mechanics approaches (i.e., QM/MM), these methods incur a prohibitive computational expense for even short timescale simulations (e.g., 30–300 CPU days for a 20 ps trajectory of a single solvated ion)^25^. Thus, there remains no single comprehensive, self-consistent set of experimental or theoretical data describing how ions interacts with water and nonaqueous molecular solvents - a ‘one-stop-shop’ of ion-solvation, so to speak.

Herein, we report the **Ion Solv**ation **R**epository (IonSolvR) - a collection of more than 3000 distinct nanosecond-scale AIMD trajectories detailing the solvation structure of up to 55 ionic solutes in 28 different molecular solvents at various effective concentrations. We circumvent the AIMD timescale issue by using density functional tight binding (DFTB)^33^, a quantum chemical method derived from generalised gradient approximation density functional theory (GGA-DFT). A number of prior studies have demonstrated the reliability of this approach for studying solvation in aqueous^34^ and nonaqueous solvent environments^14,35,36^, and other complex solvent environments such as deep eutectic solvents^37,38^ and ionic liquids^39,40^. We verify the use of this method, and the utility of the IonSolvR repository by comparing ionic solution properties with experimental data. IonSolvR is open-access and can be found at https://ionsolvr.newcastle.edu.au^41^.

## Methods

All data was generated using the DFTB+ software package (v. 19.1)^33^. Initial geometries for all MD trajectories consisted of random ensembles of solvent and solute molecules generated using the packmol^42^ package. MD simulations were performed based using 3^rd^ order density functional tight binding (DFTB3)^43^, which was computed on-the-fly at each timestep using the 3ob-3-1 parameter^44–46^ set. Grimme’s D3 dispersion^47,48^ with Becke-Johnson^49,50^ dampening (i.e., D3(BJ)) was included throughout all simulations. Charge mixing was configured with the Broyden method^51,52^. All MD trajectories were performed using constant volume & temperature dynamics (i.e., NVT ensemble) via a Nosé^53^-Hoover^54^ chain^55,56^ (NHC) thermostat (chain-length = 3) set to 300 K with a coupling constant of 1000 cm^−1^. Solvent densities were held at the experimental density of the pure solvent throughout all simulations (see Table S1 in Supplementary Information). Periodic boundary conditions (PBC) were enforced on all trajectories (cubic unit cell), with charges handled via particle mesh Ewald summation^57^. All MD trajectories were iterated using a timestep of 1 fs, with coordinates and relevant information recorded every 10 fs. MD trajectories are up to 1 ns in length; each MD trajectory in the IonSolvR therefore consists of 100,000 individual MD ‘frames’. The data contained in the IonSolvR currently represents more than 2 M CPU hours.

## Data Records

IonSolvR includes up to 55 solutes in 28 molecular solvents at 4 effective concentrations, constituting more than 3000 distinct MD trajectories in total (the physical size of the data in the repository is > 1.5 TB), see Table 1. All data within IonSolvR can be freely accessed via https://ionsolvr.newcastle.edu.au^41^. Repository records can be accessed via the website interface, or directly via command line programs (e.g., wget). Examples of how to use the wget function to download single and multiple trajectories are provided via the web interface. Individual records within IonSolvR correspond to an MD simulation of a user-specified solute (individual ion or ion pair) in a user-specified solvent of a user-specified size (i.e., number of solvent molecules), and consist of single zip files containing a complete MD trajectory in Cartesian coordinates (*.xyz* file format), a plain text file (*.out* file format) containing the energy and temperature information of the MD simulation, and a folder containing the all data for the final picosecond of the MD simulation produced by DFTB+. The latter includes the DFTB+ input file (*dftb_hsd.in*) used to generate the MD trajectory and the input geometry including PBC lattice vectors (*.gen* file format), enabling the simulation to be restarted from the final structure provided in the record. Each *.xyz* trajectory file also includes atomic charges (via total valence electron populations) and nuclear velocities (Å/ps) at each reported MD timestep, thereby enabling electronic/velocity response analyses, for instance. We note that the inclusion of charges also potentially enables the refinement of empirical point charges in classical MD force fields. The Slater-Koster parameter files required to run the simulation are not included in IonSolvR; they are freely available at https://dftb.org/parameters/download.

**Table 1:** Solvents and solutes included in IonSolvR (repository acronyms are provided in parentheses).

Solvents			Solutes				
Water (water)	1,2-dichloroethane (EDC)	Ethanol (EtOH)	CH_3_COO^−^ (acetate)	F^−^ (F)	I_3_^-^ (I3)	Na^+^ (Na)	NO_2_^−^ (NO2)
2-propanol (2PrOH)	Acetonitrile (MeCN)	Formamide (FA)	Br^−^ (Br)	HCOO^−^ (formate)	K^+^ (K)	NaBr (NaBr)	NO_3_^−^ (NO3)
Acetone (ACE)	Methanol (MeOH)	Glycerol (glycerol)	C_3_H_7_COO^-^ (butanoate)	C(NH_2_)_3_^+^ (guanidinium)	KBr (KBr)	NaCl (NaCl)	O^2−^ (O)
Benzyl alcohol (benzyl_alcohol)	Ammonia (NH3)	Hexane (hexane)	Ca^2+^ (Ca)	H^+^ (H + )	KCl (KCl)	NaF (NaF)	OCN^−^ (OCN)
Butanol (BuOH)	*N*-methylacetamide (NMA)	Hexamethylphosphoramide (HMPT)	CCl_3_COO^−^ (CCl3COO)	H^−^ (H)	KF (KF)	NaI (NaI)	OH^−^ (OH)
Diethylether (DEE)	N-methylformamide (NMF)	1-propanol (PrOH)	CF_2_ClCOO^−^ (CF2ClCOO)	H_2_PO_4_^−^ (H2PO4)	KI (KI)	N(C_2_H_5_)_4_^+^ (N2CH5_4)	C_4_H_9_COO^−^ (pentanoate)
Dimethylacetamide (DMA)	*N*-methyl-2-pyrrolidinone (NMPy)	Pyridine (Py)	CF_3_COO^−^ (CF3COO)	H_3_O^+^ (H3O)	Mg^2+^ (Mg)	N(C_3_H_7_)_4_^+^ (NC3H7_4)	PO_4_^3−^ (PO4)
Dimethylformamide (DMF)	Propylene carbonate (PC)	Trifluoroethanol (TFE)	Cl^−^ (Cl)	HCO_3_^−^ (HCO3)	MgCl_2_ (MgCl2)	N(C_4_H_9_)_4_^+^ (NC4H9_4)	C_2_H_5_COO^−^ (propionate)
Dimethylsulfoxide (DMSO)	Nitrobenzene (PhNO2)	Toluene (TOL)	ClO_4_^−^ (ClO4)	HPO_4_^2−^ (HPO4)	MgO (MgO)	N(CH_3_)_4_^+^ (NCH3_4)	SO_4_^2−^ (SO4)
	Ethylene glycol (EG)		CO_3_^2−^ (CO3)	HSO_4_^−^ (HSO4)	MgSO_4_ (MgSO4)	NCS^−^ (NCS)	TFSI (bistriflamide)
			CN^−^ (cyanide)	I^−^ (I)	N_3_^−^ (N3)	NH_4_^+^ (NH4)	Zn^2+^ (Zn)

The repository includes up to 6 simulation sizes for each combination of solvent and solute.

## Technical Validation

The performance of the DFTB method (and DFTB3 in particular^43^) is well established across a wide range of applications, including biochemistry^44–46,58–60^, solvation^13,37,38,61^, condensed phases^34,62^, electrolytes and ionic liquids^39,40^, deep eutectic solvents^38^, noncovalent interactions^63^, light-harvesting^64,65^ and electronic coupling processes^66^. We therefore do not seek to validate the performance of the DFTB method here. The technical validation of IonSolvR records instead address five salient factors – (1) the choice of DFTB parameter set, (2) the effect of effective concentration (i.e., PBC unit cell size), (3) the choice of MD integrator time step, (4) the effect of the MD thermostat coupling constant and (5) the inclusion or exclusion of a counterion. Each factor is discussed below.

**Fig. 1:**
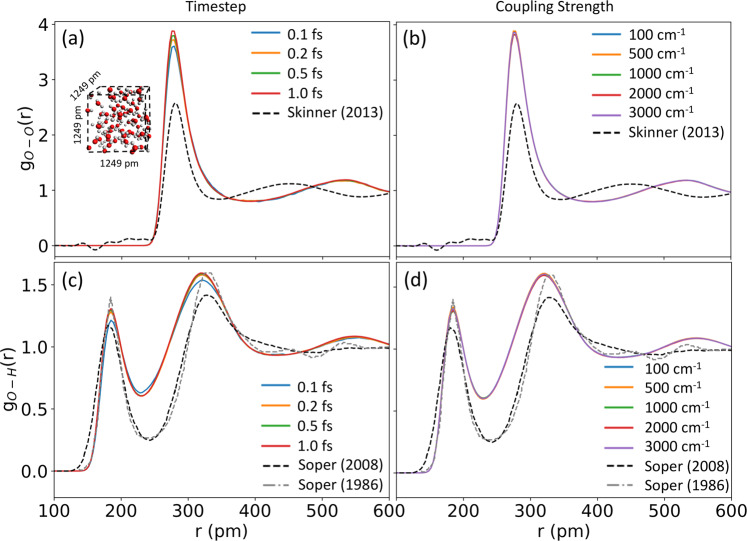
Structure of bulk water obtained from a 1249 × 1249 × 1249 pm^3^ (/65 water molecule) PBC unit cell (*ρ* = 0.99707 g·cm^-3^) using DFTB3-D3(BJ)/3ob-3-1 as a function of (**a**,**c**) timestep (fs) and (**b**,**d**) NHC coupling strength. Note that supplied data in IonSolvR use a consistent timestep of 1 fs and NHC coupling strength of 1000 cm^−1^. Structure is gauged here via the intermolecular (**a**,**b**) O-O and (**c**,**d**) O-H radial distribution functions. Experimental O-O RDFs from Skinner *et al*.^76^ and O-H RDFs from Soper *et al*.^77,78^. Sampling is performed after a 20 ps equilibration period.

IonSolvR records were generated using the 3ob DFTB parameter set, as opposed to 3obw parameter set^34^, so that each record in the repository is produced with a consistent protocol (i.e., while the 3obw parameters arguably reproduce the experimental structure of room temperature liquid water more accurately^34^, this is not guaranteed for the nonaqueous solvents included here). DFTB3/3ob has previously been studied in relation to water structure, dynamics and energetics^34,67^, with the O-H radial distribution functions reliably reproducing experimental data^34^. This agreement is evident in Fig. 1, which also demonstrates that the predicted structure of bulk water using the IonSolvR DFTB protocol is sufficiently robust with respect to both the choice of MD integrator timestep and NHC coupling parameter. The accuracy of IonSolvR records for bulk methanol, formamide, propylene carbonate and glycerol is demonstrated via comparison to experimental and ab initio data in Supporting Information (Figs. S1-S4). Perhaps the strictest test of the DFTB3-D3(BJ)/3ob-3-1 method employed here is the prediction of hydration free energy, $\Delta {G}_{{\rm{hyd}}}$, i.e. the free energy change associated with the dissolution of a solute in water. Since a MD trajectory in the NVT ensemble samples the free energy surface directly, IonSolvR enables the direct prediction of $\Delta {G}_{{\rm{hyd}}}$ via Hess’ law,

1 $$\Delta {G}_{{\rm{hyd}}}({\rm{X}}) \sim \left\langle \Delta G({{\rm{X}}}_{({\rm{aq}})})\right\rangle -\left[\left\langle \Delta G({{\rm{X}}}_{({\rm{g}})})\right\rangle +\left\langle \Delta G({{\rm{H}}}_{2}{\rm{O}})\right\rangle \right]$$

where 〈 〉 indicate time-averaging. Complete computational details are provided in Supporting Information. Fig﻿. 2 compares hydration free energies of common cations and anions predicted using Eq. (1) using IonSolvR records, with experimental values. This figure demonstrates that, in general, DFTB3-D3(BJ)/3ob-3-1 provides a reliable description of the hydration energy of the solutes considered in the IonSolvR database.

**Fig. 2:**
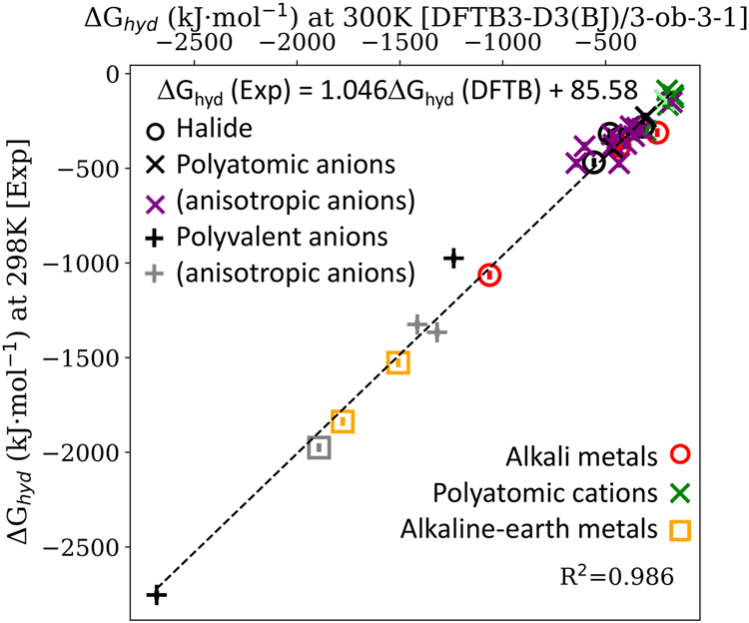
Gibbs free energies of ion hydration calculated using Eq. (1) using IonSolvR trajectories with 64 water molecules, compared to experimental values^68^. X-axis error bars indicate the standard error in the simulated $\Delta {{\rm{G}}}_{{\rm{hyd}}}\left({\rm{X}}\right)$ value. Note that the line of best fit equation here accounts for the effective concentration dependence incurred by using 64 water molecule trajectories.

**Fig. 3:**
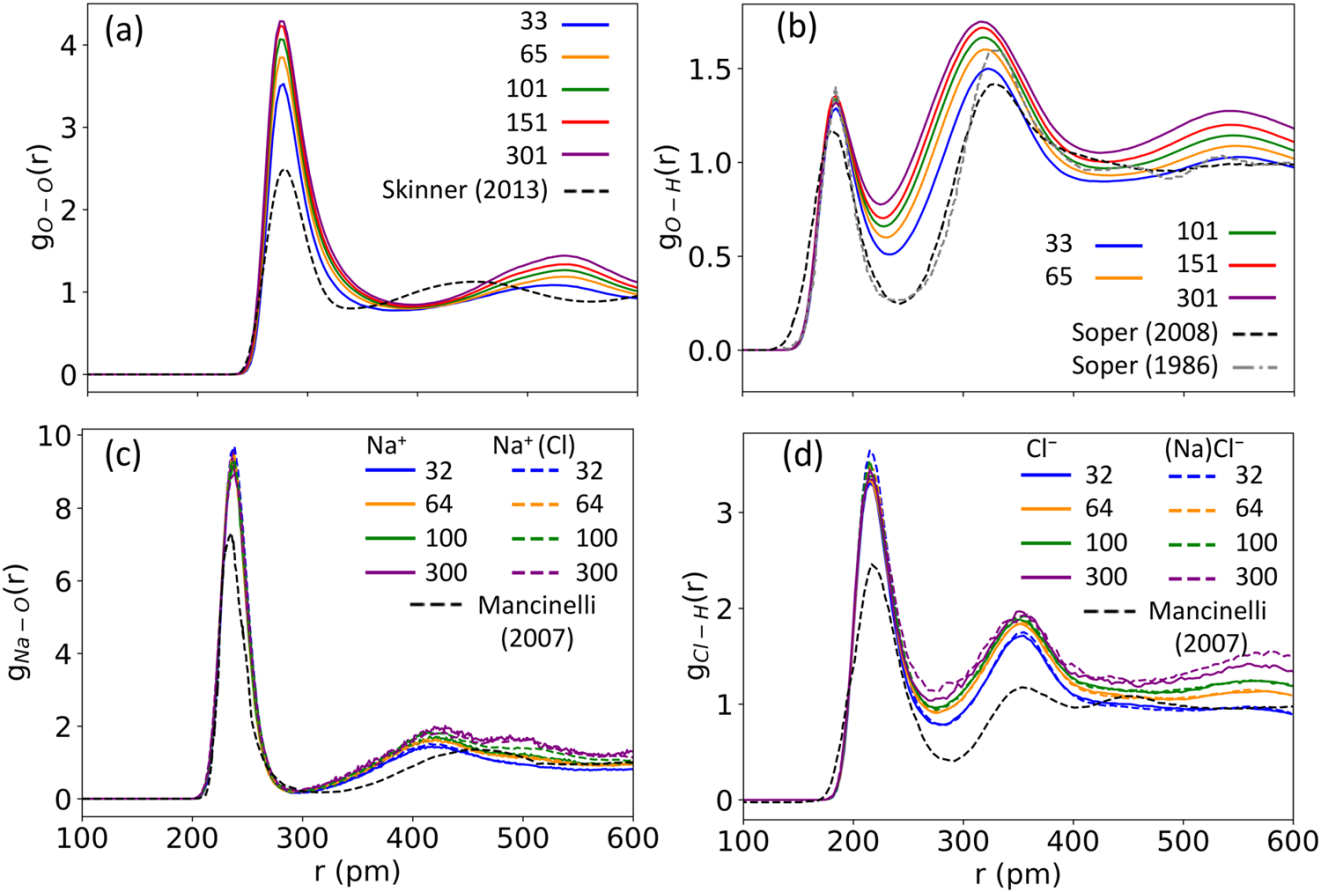
The influence of effective concentration on ion solvation in (**a**,**b**) bulk water (simulation parameters as in Fig. 1) and (**c**,**d**) aqueous NaCl solution, and solvated Na^+^ and Cl^−^ ions (based on the number of water molecules in the PBC unit cell and the experimental density of 0.997 g∙cm^−3^). Structure is gauged here via the intermolecular (**a**) O-O, (**b**) O-H, (**c**) Na-O and (**d**) Cl-H radial distribution functions. Experimental data from Skinner *et al*.^76^, Soper *et al*.^77,78^ and Mancinelli *et al*. at an effective ion:water concentration of 1:83^17^. Sampling is performed after a 20 ps equilibration period.

Due to the computational expense of DFTB, each IonSolvR trajectory was initiated at 300 K (the target ensemble temperature) without prior equilibration. The equilibration period is therefore included in the IonSolvR record itself. This does not adversely influence the description of the ion solvation structure, as is demonstrably evident from Figs. 1, 3, S1-S4 etc., since each trajectory achieves the target NVT ensemble well within 20 ps, in general (and in some cases over much shorter timescales), while the sampling we report is performed after this period. Data validating this equilibration period for select IonSolvR records is provided in Fig. S6, and the Python utility script used to perform this validation is provided to the user at https://ionsolvr.newcastle.edu.au/guides.html.

Effective concentration is achieved in IonSolvR by varying the size of the PBC unit cell. For instance, NaCl-water simulations with 32, 64, 100 or 300 water molecules, and PBC lattice vectors with lengths 1020 pm, 1260 pm, 1460 pm and 2090 pm, respectively, correspond to effective concentrations of 1.72, 0.87, 0.55 and 0.19 mol∙L^−1^, respectively (using the solvents’ experimental bulk density, see Table S1). Considering the range of experimentally available^15^ coordination numbers for Na^+^ and Cl^−^ in aqueous solutions (4–8 and 3.9–8.2 for Na^+^ and Cl^−^ respectively), the simulated effective concentrations show only subtle effects on the coordination number consistent with trends observed from neutron diffraction data reported by Mancinelli *et al*.^17^, in that the coordination number (CN) increases with decreasing concentration (Table 2).

**Table 2:** The effect of the effective concentration and the presence of a counterion on the ion’s coordination number in water.

# Solvent molecules	Effective conc. [M]	Ion-Solvent Coordination Number			
		Na^+^ (lone)	Na^+^ (NaCl)	Cl^−^ (lone)	Cl^−^ (NaCl)
32	1.72	6.31 ± 0.19	6.45 ± 0.26	6.53 ± 0.44	6.94 ± 0.44
64	0.87	6.42 ± 0.24	6.57 ± 0.24	6.91 ± 0.52	7.16 ± 0.58
100	0.55	6.52 ± 0.25	6.75 ± 0.24	7.16 ± 0.50	7.30 ± 0.55
300	0.19	6.86 ± 0.19	6.83 ± 0.21	7.13 ± 0.37	7.85 ± 0.31
**Exp**.^17^					
10	5.55		4.5 ± 1.4		5.3 ± 1.5
17	3.27		4.6 ± 1.4		5.3 ± 1.5
40	1.39		5.1 ± 0.9		5.9 ± 1.1
83	0.67		5.3 ± 0.8		6.0 ± 1.1

The simulated CN values and uncertainties are calculated from the average and standard deviations obtained via window sampling (10 ps windows), excluding the first 10 ps.

Records in IonSolvR use a set of fixed ion:solvent molecule ratios for all solvents, as opposed to a fixed PBC unit cell volume, since the former is arguably the more relevant factor for understanding ion solvation. The consequence of this choice is that, for smaller solvent molecules, such as water, lone ions are ‘closer’ to their periodic images in the PBC. One might expect that including the counterion to have a charge neutral system becomes important for small box sizes to avoid an infinite summation of charge. However, Fig. 3 and Table 2 shows that the inclusion or exclusion of a counterion for Na^+^ and Cl^−^ ions in these simulations is negligible in terms of their individual hydration structures for each effective concentration (i.e., the unit cell size governs the distance between the ion in the unit cell and its periodic images). So, while some common salts that include both cation and anion (e.g., NaCl, MgSO_4_) are provided in IonSolvR, predominantly the records consist of lone ions surrounded by solvent molecules – i.e., in the absence of a counterion. IonSolvR’s ability to describe ion solvation with no counterion present enables a broader range of solvents to be investigated without requiring the full matrix of cation-anion combinations^68^.

## Usage Notes

Guides to downloading specific trajectories are available at https://ionsolvr.newcastle.edu.au/guides.html. MD trajectories are provided in .*xyz* format and include both the Cartesian coordinates and velocities of the ensemble at each frame. Trajectory files can be quantitatively analysed by software such as TRAVIS^69,70^, MDAnalysis^71^ or MDTraj^72^ and visualised with programs such as VMD^73^, molden^74^ or Avogadro^75^. PBC lattice vectors are provided in the *.gen* geometry file for each trajectory, to enable wrapping/unwrapping of PBC coordinates, if necessary.

## Supplementary information

Supplementary Information

## Data Availability

All data contained in the IonSolvR database was generated with the DFTB+ program (v. 19.1)^33^. This code is freely available via the DFTB+ website (https://dftbplus.org/download/dftb-stable) and GitHub (https://github.com/dftbplus/dftbplus) via the GNU Lesser General Public Licence (LGPL-3). The 3ob-3-1 parameter set^44–46^ can be freely downloaded from the dftb.org website (https://dftb.org/parameters/download).
