# Supplementary material for: A quantum chemical molecular dynamics repository of solvated ions
Source: Sci Data. 2022 Jul 21;9:430. doi: 10.1038/s41597-022-01527-8 (PMC9304403; doi:10.1038/s41597-022-01527-8)
Supplement: Supplementary file 1 — Supplementary Information [file 41597_2022_1527_MOESM1_ESM.pdf]

A Quantum Chemical Molecular Dynamics Repository of Solvated Ions

Kasimir P. Gregory,[1,2] Gareth R. Elliott,[1] Erica J. Wanless,[1] Grant B. Webber,[3] Alister J. Page*[1]

1. Discipline of Chemistry, School of Environmental & Life Sciences, University of Newcastle, Callaghan NSW 2308, Australia
2. Department of Materials Physics, Research School of Physics, Australian National University, Canberra, ACT 0200, Australia
3. Discipline of Chemical Engineering, School of Engineering, University of Newcastle, Callaghan NSW 2308, Australia
**Corresponding author: Alister J. Page (alister.page@newcastle.edu.au)*

Supporting Information

Table of Contents

Table S1	2
Figure S1	3
Figure S2	4
Figure S3	5
Figure S4	6
Gibbs hydration free energy – Computational Details	7
Figure S5	7
Figure S6	8
References	9

Table S1. Experimental solvent densities used in IonSolvR

Solvent	Density (g/mL)	Ref.	Solvent	Density (g/mL)	Ref.
Water	0.99707	1,2	Formamide	1.134	1
Methanol	0.791	1	N-methylformamide	1.003	3
Ethanol	0.789	1	N-methylacetamide	0.95	1
1-propanol	0.803	2	Dimethylformamide	0.9445	1
2-propanol	0.785	2	Dimethylacetamide	0.937	1
Butanol	0.8095	1	Acetonitrile	0.786	1
Trifluoroethanol	1.3834	1	Ammonia	0.682*	4
Benzyl alcohol	1.04	1	Pyridine	0.982	1
Ethylene glycol	1.115	1	*N*-methyl-2-pyrrolidinone	1.028	1
Glycerol	1.261	1	Nitrobenzene	1.2037	1
Diethylether	0.713	1	Dimethylsulfoxide	1.092	1
Acetone	0.7845	1	Hexamethylphosphoramide	1.03	1
Propylene carbonate	1.204	1	Toluene	0.867	1
1,2-dichloroethane	1.245	1	hexane	0.6606	1

* Density value of ammonia taken at temperature of 239.8K.

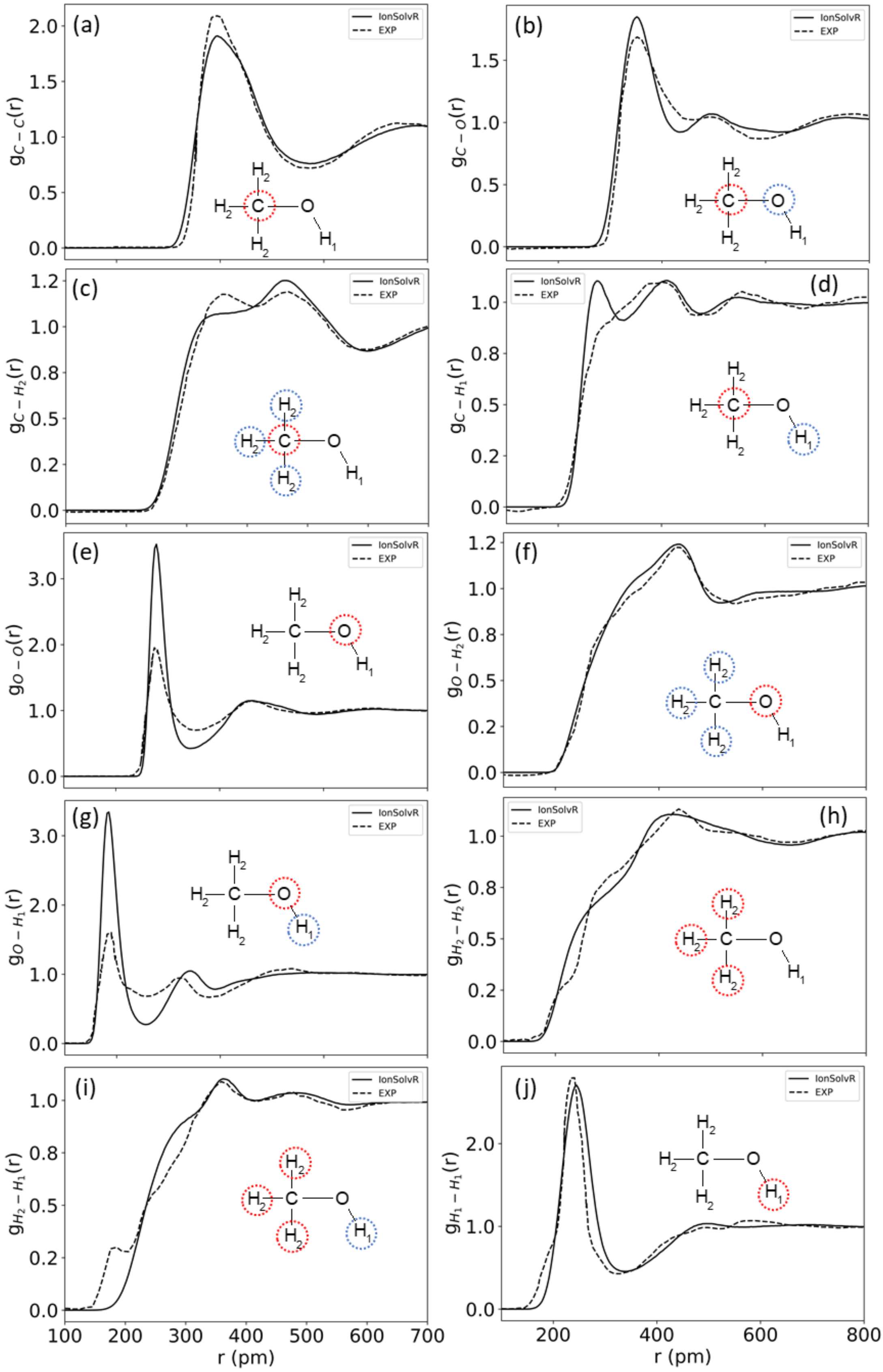

Fig. S1: Structure of bulk methanol obtained from a 1635 × 1635 × 1635 pm^3 (/65 methanol molecules) PBC unit cell (ρ = 0.791 g·cm^{-3}) using DFTB3-D3(BJ)/3ob-3-1. Experimental RDFs from pulsed neutron diffraction with isotope substitution on hydroxyl hydrogens experimental RDFs by Yamaguchi *et al.*[5]

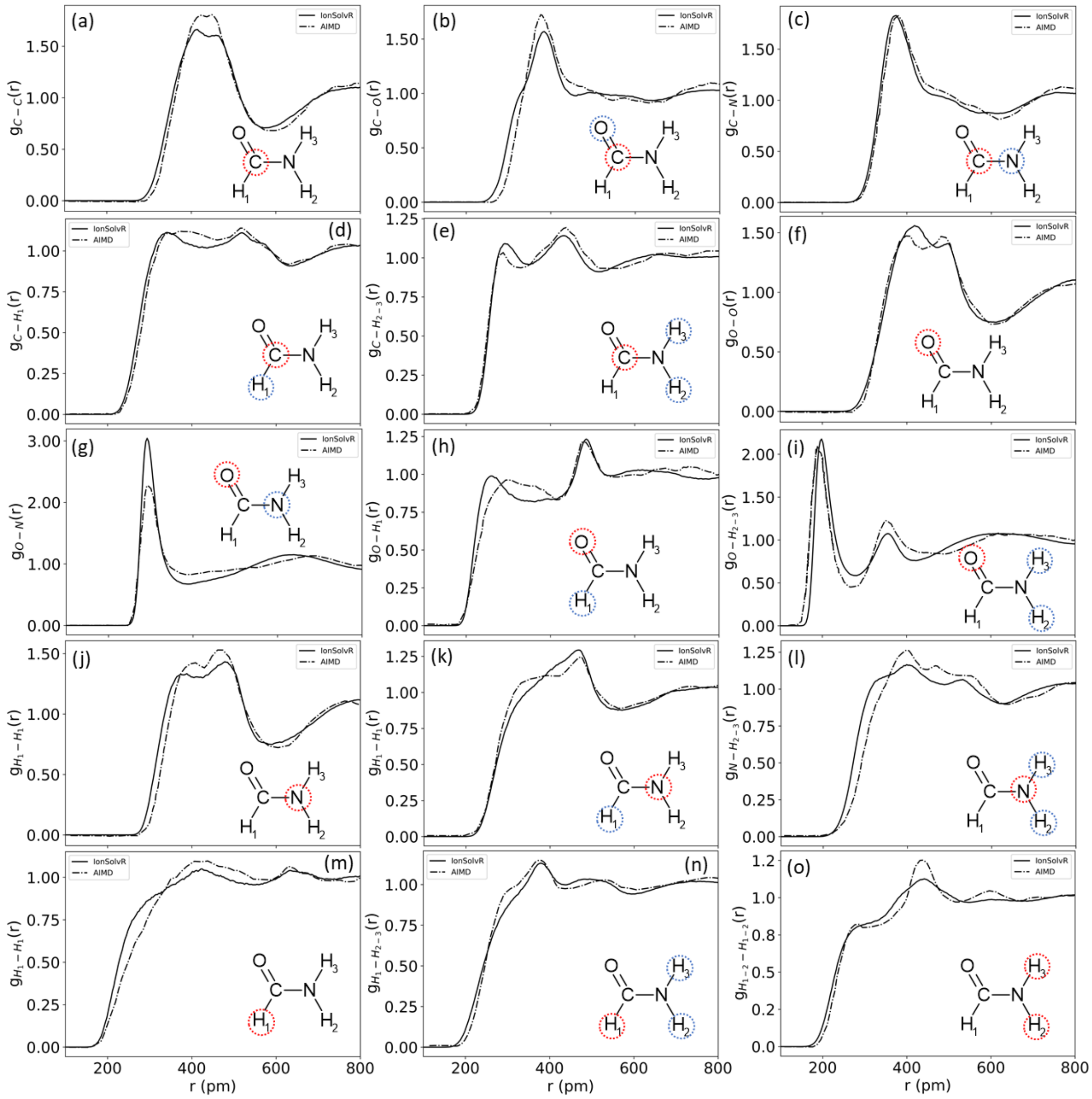

Fig. S2: Structure of bulk formamide obtained from a 1624 × 1624 × 1624 pm^3 (/65 formamide molecules) PBC unit cell (ρ = 1.134 g·cm^{-3}) using DFTB3-D3(BJ)/3ob-3-1. PBE RDFs of deuterated (for computational speedup) formamide by Tsuchida.[6]

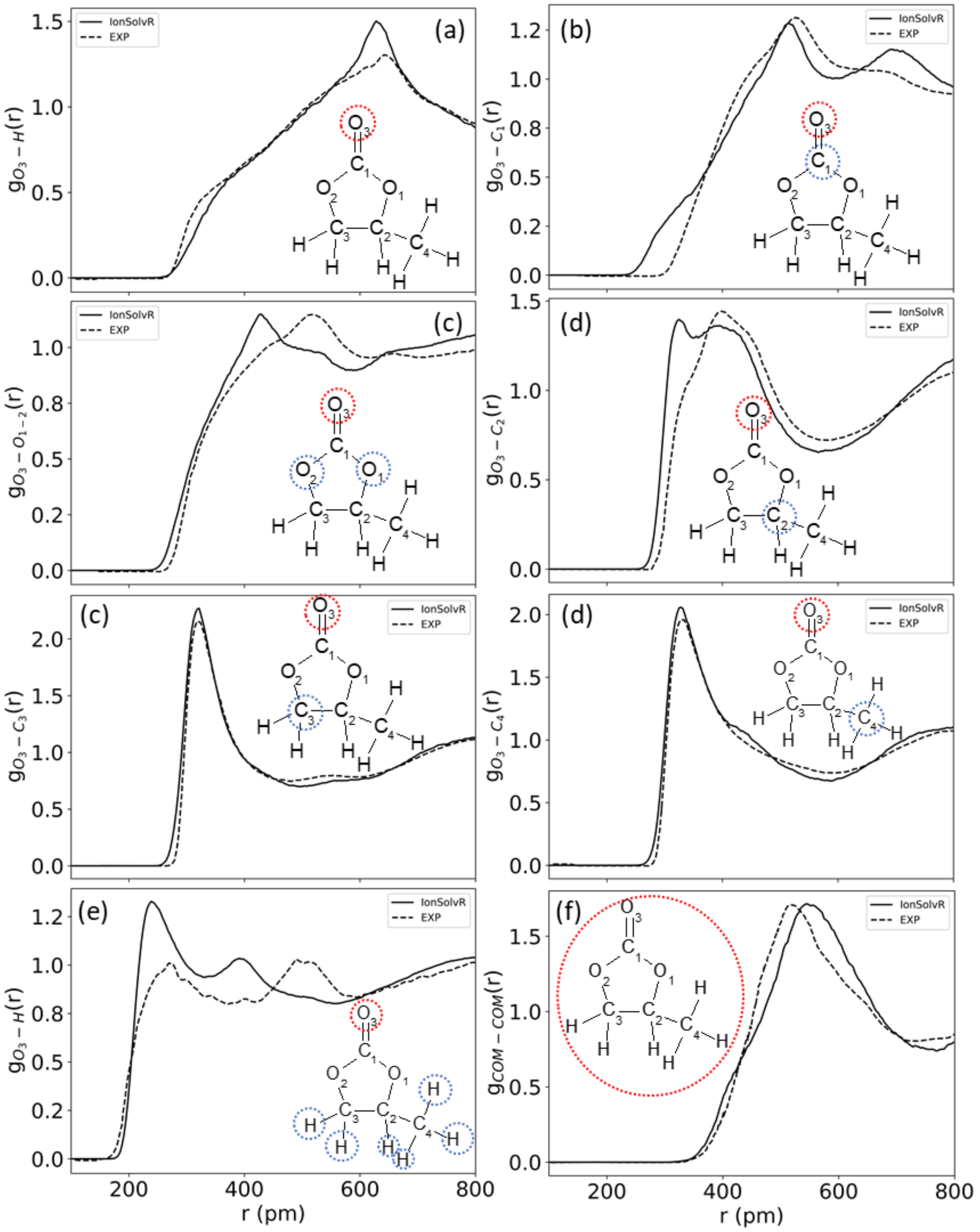

Fig. S3: Structure of bulk propylene carbonate obtained from a 1669 × 1669 × 1669 pm^3 (/33 propylene carbonate molecules) PBC unit cell (ρ = 1.2041 g·cm^{-3}) using DFTB3-D3(BJ)/3ob-3-1. Experimental RDFs from neutron diffraction with isotropic substitution by Delavoux *et al*.[7]

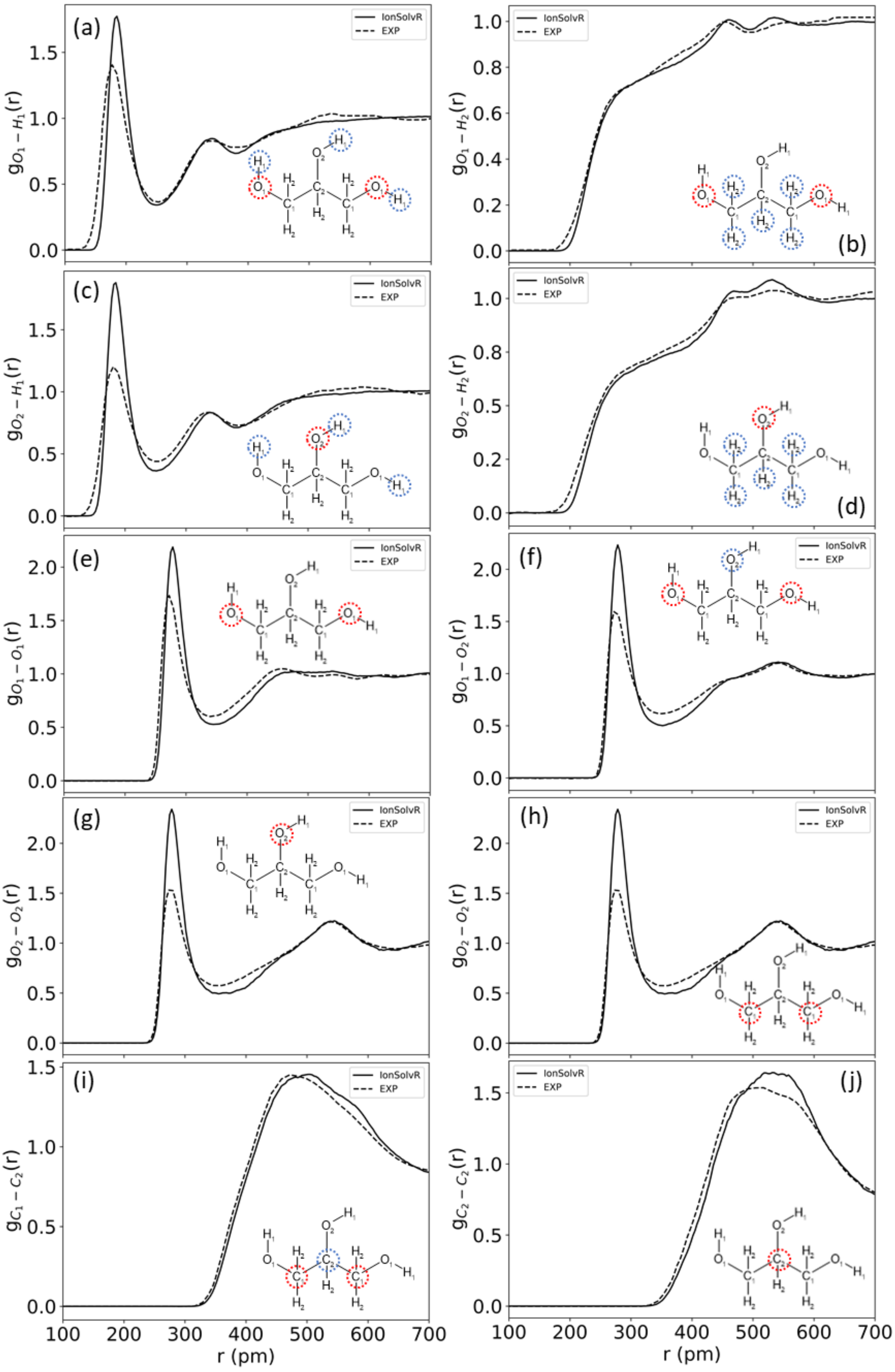

Fig. S4: Structure of bulk glycerol obtained from a 1587 × 1587 × 1587 pm^3 (/33 glycerol molecule) PBC unit cell (ρ = 1.261 g·cm^{-3}) using DFTB3-D3(BJ)/3ob-3-1. Experimental RDFs from. neutron diffraction coupled with hydrogen/deuterium isotopic substitution by Towey *et al.*[8]

Gibbs hydration free energy – Computational Details

$\langle \Delta G(\mathrm{X}_{(aq)}) \rangle$ was obtained directly from the IonSolvR trajectories of solute X in 64 water molecules, by averaging the Gibbs free energy over 1 ns. Similarly, $\langle \Delta G(\mathrm{H_2O}) \rangle$ was obtained directly from the IonSolvR trajectory of pure water, by averaging the Gibbs free energy over 1 ns. Given the latter simulation consists of a single water molecule 'solvated' with another 64 water molecules, $\langle \Delta G(\mathrm{H_2O}) \rangle$ is multiplied by 64/65 to approximate $\langle \Delta G(\mathrm{H_2O}) \rangle$ of 64 water molecules. The accuracy of this approximation is demonstrated by Fig. S5, which shows the the energy per water molecule in 32, 64, 100 and 150 water molecule trajectories. For the 65 water molecule box the correction adopted here induces a constant error of 1.86 kJ/mol in quoted $\langle \Delta G(\mathrm{H_2O}) \rangle$ values, which is included in the error bars in Fig. 2. To calculate $\langle \Delta G(\mathrm{X}_{(g)}) \rangle$, an MD simulation of the lone solute in a vacuum was performed for 1 ps at 300K and the total energy averaged across the entire trajectory. This allows for conformational sampling of configurationally complex solutes such as the tertiary ammonium cations. Given the number of timesteps sampled for these calculations, the standard error is considered negligble.

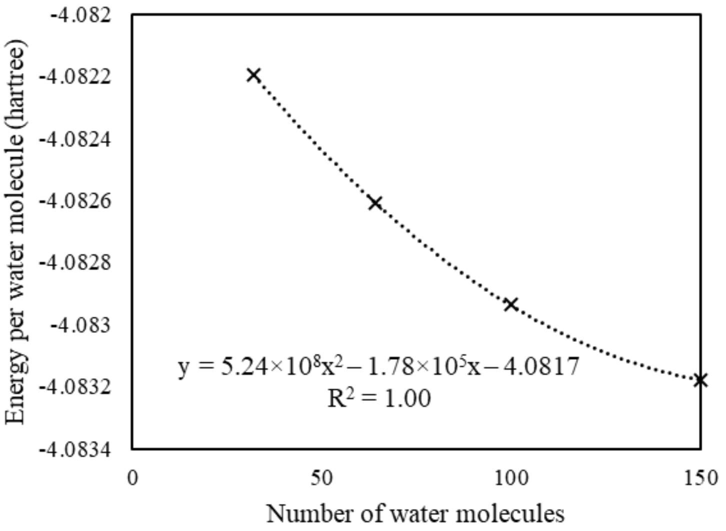

Fig. S5: The energy per water molecule dependence on the number of water molecules in periodic unit cells employed in the IonSolvR database. The quadratic polynomial fit has been used here to estimate the error associated with using the 64/65 ratio for the solvent simulation energy to be used in the $\Delta G_{hyd}(\mathrm{X})$ calculations.

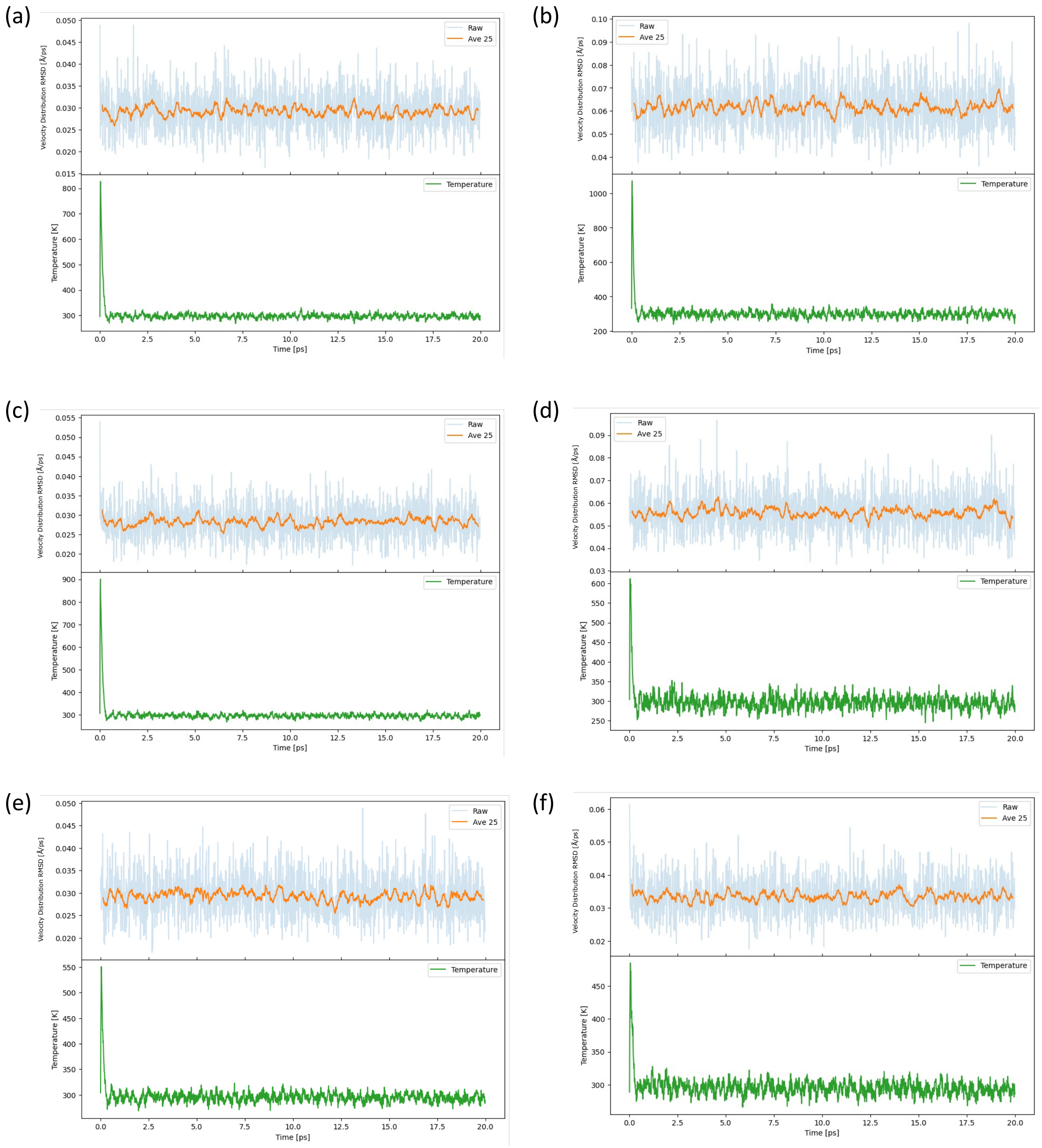

Fig. S6: Validation of NVT equilibration in IonSolvR trajectories. Each image shows the instantaneous MD temperature of the trajectory in the bottom panel, and the instantaneous and moving-average velocity distribution RMSDs. The latter are based on the atomic velocity distributions in consecutive MD frames. A constant RMSD indicates that Maxwell-Boltzmann statistics are conserved across the MD trajectory (i.e. that the NVT ensemble is conserved). In general, the NHC thermostat achieves equilibration within 20 ps. Trajectories have been validated in terms of periodic unit cell size (i.e. number of solvent molecules), for both archetypal 'simple' and 'configurationally complex' solutes (e.g. Cl^- and $N(C_4H_9)^+$) and solvents (e.g. water, ethylene glycol). To perform this analysis, a python script is made available at https://ionsolvr.newcastle.edu.au. (a) Cl^-, 300 waters. (b) Cl^-, 64 waters. (c) $N(C_4H_9)^+$, 300 waters. (d) $N(C_4H_9)^+$, 64 waters. (e) Cl^-, 64 ethylene glycols. (f) $N(C_4H_9)^+$, 64 ethylene glycols.

References

1. Lide, D. R. *CRC handbook of chemistry and physics*. vol. 87 (CRC press, 2006).
2. W.M. Haynes. *CRC handbook of chemistry and physics*. vol. 97 (CRC press, 2016).
3. PubChem Compound Summary for CID 31254, N-Methylformamide. *National Center for Biotechnology Information (2021)* https://pubchem.ncbi.nlm.nih.gov/compound/N-Methylformamide.
4. PubChem Compound Summary for CID 222, Ammonia Title. *National Center for Biotechnology Information (2021).* https://pubchem.ncbi.nlm.nih.gov/compound/Ammonia.
5. YAMAGUCHI, T., HIDAKA, K. & SOPER, A. K. The structure of liquid methanol revisited: a neutron diffraction experiment at −80 °C and +25 °C. *Mol. Phys.* **96**, 1159–1168 (1999).
6. Tsuchida, E. Ab initio molecular-dynamics study of liquid formamide. *J. Chem. Phys.* **121**, 4740–4746 (2004).
7. Delavoux, Y. M., Gilmore, M., Atkins, M. P., Swadźba-Kwaśny, M. & Holbrey, J. D. Intermolecular structure and hydrogen-bonding in liquid 1,2-propylene carbonate and 1,2-glycerol carbonate determined by neutron scattering. *Phys. Chem. Chem. Phys.* **19**, 2867–2876 (2017).
8. Towey, J. J., Soper, A. K. & Dougan, L. The structure of glycerol in the liquid state: a neutron diffraction study. *Phys. Chem. Chem. Phys.* **13**, 9397–9406 (2011).
9. Marcus, Y. *Ions in Solution and their Solvation*. (John Wiley & Sons, 2015).
